# Dietary fat and corticosterone levels are contributing factors to meal anticipation

**DOI:** 10.1152/ajpregu.00308.2015

**Published:** 2016-01-27

**Authors:** Sara Namvar, Amy Gyte, Mark Denn, Brendan Leighton, Hugh D. Piggins

**Affiliations:** ^1^Faculty of Life Sciences, University of Manchester, Manchester, United Kingdom; and; ^2^AstraZeneca Research and Development, Mereside, Alderley Park, Macclesfield, United Kingdom

**Keywords:** food entrainable oscillator, corticosterone, meal anticipation, high-fat diet

## Abstract

Daily restricted access to food leads to the development of food anticipatory activity and metabolism, which depends upon an as yet unidentified food-entrainable oscillator(s). A premeal anticipatory peak in circulating hormones, including corticosterone is also elicited by daily restricted feeding. High-fat feeding is associated with elevated levels of corticosterone with disrupted circadian rhythms and a failure to develop robust meal anticipation. It is not clear whether the disrupted corticosterone rhythm, resulting from high-fat feeding contributes to attenuated meal anticipation in high-fat fed rats. Our aim was to better characterize meal anticipation in rats fed a low- or high-fat diet, and to better understand the role of corticosterone in this process. To this end, we utilized behavioral observations, hypothalamic c-Fos expression, and indirect calorimetry to assess meal entrainment. We also used the glucocorticoid receptor antagonist, RU486, to dissect out the role of corticosterone in meal anticipation in rats given daily access to a meal with different fat content. Restricted access to a low-fat diet led to robust meal anticipation, as well as entrainment of hypothalamic c-Fos expression, metabolism, and circulating corticosterone. These measures were significantly attenuated in response to a high-fat diet, and animals on this diet exhibited a postanticipatory rise in corticosterone. Interestingly, antagonism of glucocorticoid activity using RU486 attenuated meal anticipation in low-fat fed rats, but promoted meal anticipation in high-fat-fed rats. These findings suggest an important role for corticosterone in the regulation of meal anticipation in a manner dependent upon dietary fat content.

circadian rhythms in behavior and physiology are generated by the master circadian clock located in the suprachiasmatic nucleus (SCN) of the anterior hypothalamus, as well as extra-SCN circadian oscillators present in other neural and peripheral tissues (7, 17, 22, 27, 39). The SCN communicates clock phase information to the rest of the brain and body directly through synaptic connections and indirectly through regulation of hormonal signals (5, 71). Indeed, corticosterone, a product of the adrenal gland, is under circadian control by the SCN, as well as a local adrenal clock (8, 37). Corticosterone's circadian release profile contributes to the coordination of some, but not all, circadian oscillators present in peripheral tissues (17, 78). In nocturnal rodents housed under light-dark conditions, corticosterone levels peak near the onset of night, suggesting corticosterone has a role in night phase activity when nocturnal animals become most active and consume the majority of their food (12, 66, 69). This late-day rise in corticosterone provides negative feedback to the paraventricular hypothalamus (PVH), where it acts to decrease corticotrophin-releasing factor production, and ultimately corticosterone itself (13, 66). Interestingly, some animal models of metabolic disorders, such as the genetically obese Zucker or high-fat-fed rodents have high basal levels of corticosterone and exhibit dampened circadian rhythmicity in feeding and behavior (40, 53, 79).

In rodents, SCN control of behavior and physiology can be overridden when food availability is confined to particular times of the day. For example, restriction of a daily meal to the first half of the light phase leads to the development of food-anticipatory activity (FAA) (11, 60, 61). Restricted feeding (RF) causes reorganization of daily patterns of certain genes in the hypothalamus, including the immediate-early gene, *c-fos* (a marker of neuronal activation), and circadian clock genes, such as *Period1* and *Period2* (3, 35, 72, 80). The development of FAA arises through the activation of extra-SCN timekeepers that function as food-entrainable oscillators (FEOs) (10, 31, 61). The precise location of FEOs is unclear, but studies using experimental brain lesions and genetic manipulation suggest roles for several hypothalamic areas. These include the dorsomedial (DMH) (21, 26, 43, 45, 47, 64), ventromedial (VMH) (42, 63), lateral hypothalamus (2, 58), and PVH (46) in the expression of some forms of FAA, although there are inconsistencies between studies. Additionally, several extra-hypothalamic brain regions have also been implicated in meal anticipation (57, 62, 68, 77). The current consensus is that no single brain region or peripheral tissue houses an omnipotent FEO, but rather a network of brain and peripheral sites orchestrate and contribute to meal anticipatory activity (10, 55, 69).

Exposure to RF also entrains the hypothalamic-pituitary-adrenal (HPA) axis, including the daily pattern of corticosterone, such that plasma corticosterone rises prior to presentation of the daily meal (13, 19, 32, 33, 69). This entrained corticosterone rhythm persists in the absence of meal presentation, suggesting that it is an anticipatory rather than a hunger signal (32). Despite this observation, evidence concerning the role of the HPA axis and corticosterone in FAA is somewhat conflicting. For example, adrenalectomy (ADX), prevents behavioral and physiological adaptation to RF, leading to insufficient food intake and high mortality rate (38). Further, a dose-dependent action of corticosterone on food-seeking behavior has been described (14). A more recent investigation using ADX rats demonstrated the necessity of a premeal peak of corticosterone in FAA (19). In that study, ADX rats did not display FAA, even when implanted with a corticosterone pellet. Conversely, others have shown a premeal peak in corticosterone is not required for the development of FAA (78).

Intriguingly, obesity is associated with elevated corticosterone, and treatment with the potent glucocorticoid receptor (GR) antagonist RU486 can combat obesity and related complications (29, 34, 48, 67). Rodents receiving a single daily meal rich in saturated fat show attenuated FAA, associated with a lack of meal anticipatory corticosterone, measured at a single time point (70, 75). Interestingly, the F344 rat, an animal with high basal levels of corticosterone, shows relatively low meal anticipatory wheel-running activity (18). Thus, while corticosterone is likely to be important for FAA (19), high basal levels of this hormone may impair the development and/or expression of FAA in high-fat fed rats.

The aim of the current study was to investigate the effect of dietary fat content on rhythms in physiology and ingestive behavior of rats maintained under RF relative to ad libitum-fed conditions. Specifically, we compared how rats given a high-fat diet (HFF) or regular rat chow (C) under control ad libitum (AL) or restricted feeding (RF) regimens varied in their *1*) anticipatory behavior (FAA); *2*) daily metabolic profile, as measured by indirect calorimetry of respiratory gases; and *3*) the expression of a marker of neuronal activation, c-Fos, in the DMH, PVH, and SCN. In addition, we examined the daytime profile of corticosterone under the different conditions, as well as the effect on FAA of blocking corticosterone's actions with the antagonist RU486. Our results reveal that HFF attenuates behavioral, neural, and physiological manifestations of FAA, while transient blockade of corticosterone's actions by RU486 tended to reverse these actions of the high-fat diet.

## MATERIALS AND METHODS

### Animals

Male Han Wistar rats (Harlan UK) weighing ∼240 g at the start of the study were housed in pairs. Rats were acclimatized to an ad libitum standard laboratory chow (C-AL) and maintained under 12:12-h light-dark cycle [lights on at 6 AM; where zeitgeber time (ZT) 0 = lights on and ZT12 = lights off]. Following an initial acclimatization period, half of the rats were transferred to 45% high-fat diet (Research Diets, New Brunswick, NJ) ad libitum (HFF-AL) for 3 wk. Next, half of the C-AL and half of the HFF-AL rats were subject to daily RF of their respective diets for 3 wk, to form two new groups: C-RF and HFF-RF, respectively. For RF animals, food was presented each morning at 9 AM/ZT3 (ZT0 denotes lights on, ZT12 denotes lights off) and removed 4 h later at 1 PM (ZT7), while for ad libitum-fed rats, food was continuously available. For c-Fos and corticosterone sampling (see *Immunohistochemistry* and *Quantification of Plasma Corticosterone*), restricted-fed animals were subject to an overnight fast (up to 24 h) and killed at one of three time points. Body weight and energy intake were monitored, and energy efficiency was calculated [body weight change (g)/kcal consumed]. All experiments were performed in accordance with the UK Home Office, Animals in Scientific procedures Act (Licence 40/3023).

### Characterization of Meal Anticipatory Behavior

The behavior of rats was recorded by digital video onto DVDs in the 80 min preceding the daily meal. The range of behaviors displayed during the anticipatory phase was later scored offline (*n* = 4 per group). A scoring system was devised such that the behavior expressed by each rat was noted every minute in the time before meal presentation. The number of food hopper approaches, in particular, as well as the range of behaviors expressed in the 80 min prior to delivery of the meal, were determined for each day of recording by observers blind to the treatment condition.

### Immunohistochemistry

At the end of the study, animals received an intraperitoneal injection of pentobarbital sodium (50 mg/kg) and were transcardially perfused with 4% paraformaldehyde (Sigma, Dorset, UK) at 9 AM/ZT3 (immediately prior to presentation of food in the RF animals) or ZT7 (postanticipation) in the fasted state. As previously described, 30-μm-thick coronal brain sections were collected for immunohistochemically for c-Fos (52). In brief, following a series of 0.1 M phosphate buffer (PB) washes and 0.1% H_2_O_2_ (30%, Sigma) in 0.1 M PB Triton X-100 (Sigma), sections were blocked with 1% BSA for 1 h. Sections were then incubated with c-Fos rabbit polyclonal primary antibody (Santa Cruz, Heidelberg, Germany) in blocking buffer overnight on a rotating platform at 4°C. Following a series of PB wash steps, sections were incubated for 90 min with biotinylated secondary antibodies (goat-anti-rabbit; Vector Laboratories, Burlingame, CA). After further washes, tissue was incubated with avidin-biotin-peroxidase complex (ABC: 1:400 PBS, Vectastain Elite ABC Kit, Vector Laboratories) for 90 min, before being washed, and staining was visualized using 3, 3′-diaminobenzidene and nickel chloride (Vector Laboratories). Once sections were mounted, a microscope equipped with a digital camera was used to collect ×20 magnification images of the SCN, DMH compacta (DMHc), and PVH with anatomical landmarks clearly visible to aid subsequent analysis. Total c-Fos in each nuclei of interest was then analyzed using Image-Pro Analyzer 6.3 software (Media Cybernetics, Silver Spring, MD). A minimum of four sections were analyzed per animal for each brain region of interest.

### Quantification of Plasma Corticosterone

In a separate group of animals, the four feeding groups C-AL, HFF-AL, C-RF, and HFF-RF were set up, as previously described. At the end of this study, animals were fasted and then concussed and decapitated (according to Schedule 1 UK Home Office-approved method); trunk blood was collected in EDTA tubes at ZT3, ZT7, and ZT11 and kept on ice. Plasma corticosterone was then quantified using a commercially available enzyme immunoassay kit (Immunodiagnostic Systems, Boldon, UK), according to the manufacturer's protocol.

### Analysis of Metabolism by Indirect Calorimetry

Indirect calorimetry chambers appropriate for singly housed rats and powdered diets were used to assess the metabolic rhythms of rats subject to the various feeding regimes described above. These chambers were equipped with infrared photocells able to detect general cage activity and activity at the feeding hopper. Following acclimatization and 3 wk of control or high-fat feeding (in the powdered form), both C-AL and HFF-AL rats were transferred to indirect calorimetry Oxymax cages (Columbus Instruments, OH) for 4 days to assess the effect of standard and high-fat diet on metabolism. Rats were then returned to their home cage and subject to RF of their respective powdered diets for 14 days to permit meal entrainment to establish. These singly housed rats (C-RF and HFF-RF) were then transferred to the indirect calorimetry cages, where the RF regimen continued for a further 4 days and metabolism was assessed. Several behavioral and physiological parameters were analyzed, including food intake, locomotory activity in the cage, number of food hopper visits, oxygen (O_2_), and carbon dioxide (CO_2_) consumption, as well as the respiratory quotient (RQ). RQ values above 1 indicate lipogenesis; those around 0.7 indicate lipolysis, and those between 0.7 and 1 indicate the use of a variety of substrates (20, 50, 54).

### Antagonism of Corticosterone Action Using RU486

10 mg/kg RU486 suspended in hydroxypropylmethylcellulose Tween was prepared for oral administration, while 5 mg/kg RU486 was prepared for intravenous administration by first dissolving in 5% DMSO and then made up to final volume with 95% Sorenson 5.5 buffer containing 30% hydroxypropylbetacyclodextrin. Rats were either orally dosed (10 mg/kg) or intravenously dosed (5 mg/kg) with RU486. Tail prick blood samples were collected over a period of 24 h, and the concentration of RU486 in these samples determined by liquid chromatography-tandem mass spectrometry, similar to a previously described protocol (28).

On the basis of pharmacokinetics data generated, dosing rats intravenously with 5 mg/kg RU486 at ZT7 (postfeeding period) was postulated to provide glucocorticoid receptor occupancy and antagonism for ∼16 h, permitting endogenous corticosterone action in the window just before meal presentation the next morning. Therefore, in a final study, rats were assigned to C-RF or HFF-RF schedules, as described above, and they additionally received a daily dose of 5 mg/kg RU486 or vehicle at ZT7 every day for 12 consecutive days. Behavior was recorded and scored for the 120 min preceding meal presentation.

### Statistical Analysis

Group sizes were determined on the basis of available data using internal power analysis tools at AstraZeneca. Data were analyzed in Prism version 5.04 (GraphPad, La Jolla, CA), by ANOVA (repeated measures or standard two-way ANOVA, as appropriate) with Tukey post hoc tests for multiple comparisons. All experiments were repeated to confirm observations. Statistical significance was defined as *P* < 0.05. Data are presented as means ± SE. Significance obtained by comparing RF groups with respective ad libitum controls is indicated by * symbols as described in the figure legends. Where significance was detected compared to ad libitum fed or RF rats on the counter diet the symbols # and $ have been used respectively. In the case of # and $, P values can be found in the main text.

## RESULTS

### 

#### Restricted access to high-fat diet attenuates meal anticipatory behavior and alters hypothalamic c-fos expression.

**Fig. 1.**
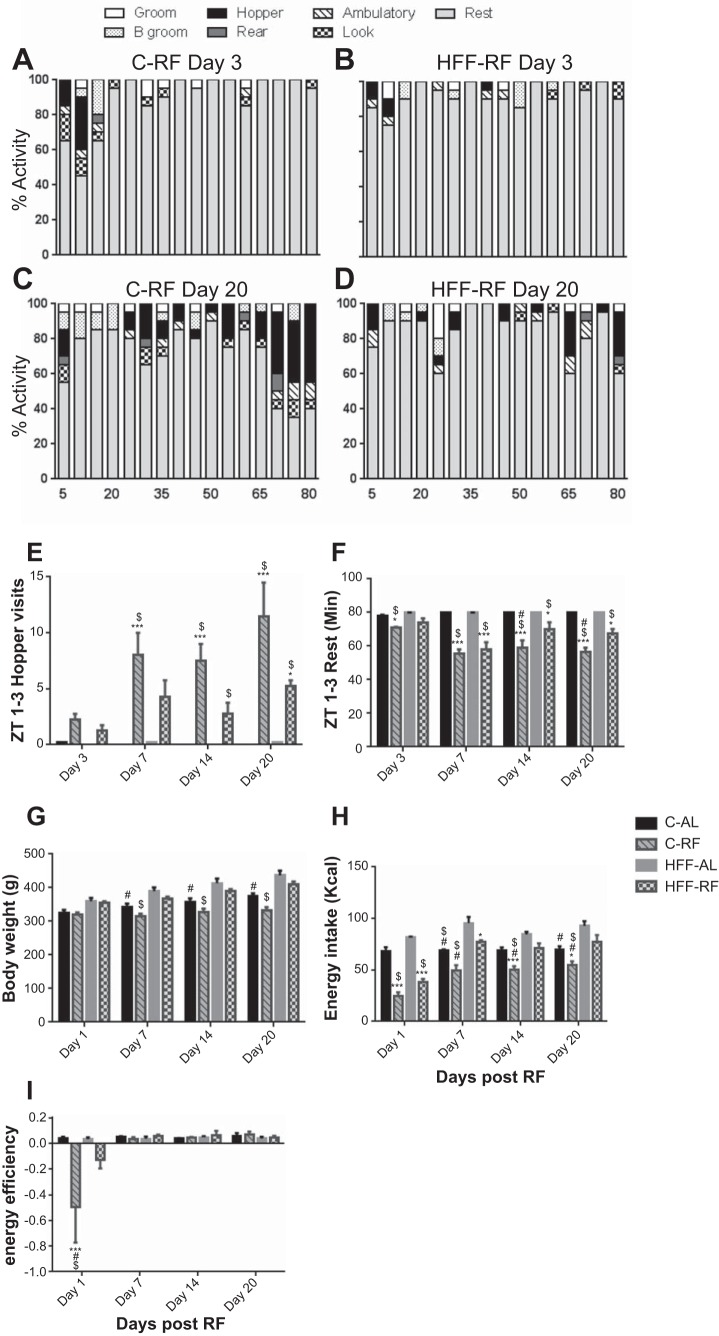
High-fat diet attenuates meal anticipation in restricted-fed rats. *A–D*: average behavioral stack plots, depicting the range of behaviors presented by restricted-fed rats (on control and high-fat diets) in the 80 min preceding the daily meal. By *day 20*, control-restricted diet fed (C-RF) animals displayed more active behaviors than high-fat diet fed (HFF-RF) counterparts. Ad libitum control rats did not exhibit active behaviors throughout the 80-min assessment epoch at any point in the study (data not shown). *E*: from *day 7* onward, C-RF rats showed significantly more food hopper visits than control C-AL animals in this 80-min assessment epoch. Similarly, HFF-RF rats exhibited meal anticipation but to a lesser extent than C-RF rats. *F*: C-RF and to a lesser extent HFF-RF animals showed significantly less resting than ad libitum-fed counterparts. *G*: C-AL rats were consistently lighter than high-fat diet fed-ad libutum (HFF-AL) animals, while C-RF rats were lighter than rats of both HFF groups. *H*: restricted feeding significantly reduced calorie consumption in C-RF rats relative to C-AL animals. From *day 1* to *day 7*, HFF-RF rats reduced calorie consumption relative to HFF-AL animals, but are comparable to controls at *day 14*. *I*: C-RF rats show a significant reduction in energy efficiency on the first day of RF. HFF-RF rats display a modest reduction in energy efficiency on the first day of restricted feeding. Energy efficiency was comparable between groups on every other subsequent day assessed. *P < 0.01, **P < 0.001, ***P < 0.0001, significant difference within diet comparisons. $Significant difference compared with HFF-RF group. #Significant difference compared with HFF-AL group. *n* = 4–8 per group.

In response to timed food access over 21 days, C-RF animals and to a lesser extent HFF-RF rats progressively increased active behaviors relative to ad libitum controls in the time preceding the daily meal (Fig. 1, *A–D*). Two-way ANOVA with repeated measures revealed a significant main effect of feeding group [*F* (3, 12) = 22.57, *P* < 0.0001], time point in the study [*F*(3, 36) = 6.250, *P* < 0.001], and a feeding group × time point interaction [*F*(9, 36) = 2.755, *P* < 0.01] on premeal anticipatory hopper visits (Fig. 1*E*). By *days 7* and *14* of the feeding regimen, C-RF rats approached the hopper significantly more frequently than both ad libitum-fed groups (Tukey test, both *P* < 0.001, Fig. 1*E*). By *day 14*, C-RF rats approached the hopper significantly more than HFF-RF rats and controls (Tukey, *P* < 0.01), highlighting the lack of anticipation in the latter mentioned group. These observations were more pronounced by *day 20*, such that C-RF rats approached the hopper significantly more frequently than both ad libitum-fed groups (Tukey, *P* < 0.0001) and HFF-RF rats (Tukey, *P* < 0.001). At *day 20*, HFF-RF animals approached the hopper significantly more frequently than the HFF-AL rats (Tukey, *P* < 0.01).

Accordingly, in response to restricted feeding, both HFF-RF and to a greater extent C-RF rats showed a progressive decrease in resting behavior. Two-way ANOVA with repeated measures revealed a significant main effect of feeding group [*F* (3, 12) = 44.22, *P* < 0.0001], time point in the study [*F* (3, 36) = 9.76, *P* < 0.001], and a feeding group × time point interaction [*F*(9, 36) = 4.26, *P* < 0.0008] on premeal resting behavior. By *day 3*, C-RF rats showed significantly less resting behavior than both ad libitum-fed groups (Tukey, *P* < 0.01). This difference became pronounced over time (Tukey tests, all *P* < 0.0001). Similarly, compared with ad libitum control animals, HFF-RF rats showed less resting behavior on *days 7*, *14*, and *20* (Tukey tests, *P* < 0.01 to < 0.0001). In agreement with the relatively high number of premeal hopper approaches observed in C-RF rats, this group showed significantly less rest than HFF-RF rats on *days 14* and *20* (Tukey tests, *P* < 0.001).

We next assessed whether the difference in the development of meal anticipation observed in C-RF and HFF-RF rats coincided with alterations in body weight and energy intake. Two-way ANOVA with repeated measures revealed a significant main effect of feeding group [*F* (3, 20) = 9.702, *P* < 0.0004], time point in the study [*F*(3, 60) = 310.5, *P* < 0.0001], and feeding group × time point interaction [*F*(9, 60) = 21.87, *P* < 0.0001] on body weight. On the first day following 3 wk of ad libitum feeding on the low- and high-fat diet, there was no statistical difference in the body weights of the four feeding groups. By *day 7* of RF, C-AL (Tukey, *P* < 0.01) and to a greater extent C-RF rats (Tukey, *P* < 0.0001) were significantly lighter than HFF-AL rats. Further, CRF-RF rats were significantly lighter than HFF-RF rats (Tukey, *P* < 0.001). This trend continued and became strengthened over the course of the study, such that by *day 20* of restricted feeding, HFF-AL rats were significantly heavier than both C-AL (Tukey, *P* < 0.001) and C-RF (Tukey, *P* < 0.0001) rats. In addition HFF-RF rats were notably heavier than C-RF rats (Tukey, *P* < 0.0001). However, no significant difference was observed between C-RF and their C-AL controls or HFF-RF and HFF-AL control rats, suggesting recovery of energy homeostasis in both experimental groups relative to corresponding controls.

We next assessed the impact of diet and feeding pattern on energy intake. Two-way ANOVA with repeated measures revealed a significant main effect of feeding group [*F* (3, 12) = 39.61, *P* < 0.0001], time point in the study [*F* (3, 36) = 37.90, *P* < 0.0001], and feeding group × time point interaction [*F*(9, 36) = 12, *P* < 0.0001] on energy intake. This was mainly due to a consistent reduction in the calories consumed by C-RF rats relative to other groups. On *days 1*, *14*, and *20* of the feeding regimen, C-RF rats consumed significantly fewer calories than other ad libitum groups (Tukey tests, all *P* < 0.0001). Conversely, HFF-RF significantly reduced food intake only on *day 1* relative to both control groups (Tukey tests, *P* < 0.0001) and *day 7* (Tukey tests, *P* < 0.01) relative to HFF-AL rats. Thus, C-RF rats were consistently calorie-restricted, with a decline in significance relative to C-AL rats by *day 20* (Tukey, *P* < 0.01), while HFF-RF rats had almost recovered calorie consumption by the end of the study relative to their HFF-AL controls. Thus, while energy homeostasis is recovered in C-RF rats, such that body weight is regulated, this is achieved with a deficit in calorie consumption. Energy efficiency describes the change in body weight occurring in response to the consumption of a given number of calories. We next quantified energy efficiency as a means to understand how well RF animals adapt to limited food access. Two-way ANOVA with repeated measures revealed a significant main effect of feeding group [*F* (3, 48) = 2.92, *P* < 0.04], time point in the study [*F* (3, 48) = 6.77, *P* < 0.0007], and feeding group × time point interaction [*F* (9, 48) = 12, *P* < 0.004] on energy efficiency. Post hoc tests revealed that on the first day of RF, the energy efficiency of C-RF rats was significantly lower than that of both ad libitum fed groups (Tukey, *P* < 0.0001), as well as HFF-RF rats (Tukey, *P* < 0.001). However, on every other day subsequently assessed, energy efficiency was comparable between the four feeding groups. This indicates that groups adapted to the experimental RF conditions, such that their energy efficiency is indistinguishable from animals under control conditions.

**Fig. 2.**
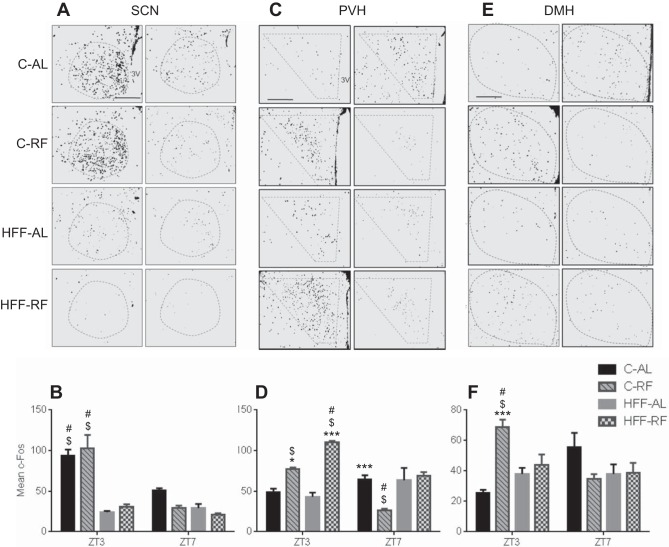
Diet-dependent variation in hypothalamic c-Fos expression. *A* and *B*: C-AL animals showed an early (zeitgeber time 3, ZT3) to mid-day (ZT7) reduction in c-Fos expression in the SCN, which was unaffected by restricted feeding (C-RF). Compared with control diet counterparts, HFF animals show reduced SCN c-Fos expression at ZT 3, which does not reduce further at ZT7. *C* and *D*: The ZT3 to ZT7 rise in c-Fos expression in the PVH seen in C-AL rats was reversed by restricted feeding (C-RF group). No apparent variation in c-Fos expression from ZT3 to ZT7 was observed in HFF-AL group. At ZT3, restricted feeding caused a significant increase in c-Fos expression in the PVH of both C-RF and HFF-RF rats relative to ad libitum animals. *E* and *F*: The ZT3 to ZT7 rise in c-Fos expression in the DMH seen in C-AL rats was reversed in response to C-RF. This observed anticipatory rise in in DMH c-Fos levels seen in C-RF rats at ZT3 is absent in HFF-RF rats. No apparent variation in c-Fos expression from ZT3 to ZT7 was observed in HFF groups. *P < 0.01, ***P < 0.0001, significant difference within diet comparison. $Significant difference compared with counterpart RF group. #Significant difference compared with counterpart AL group. *n* = 4 per group.

Although the SCN is not necessarily implicated in entrainment to timed daily RF, it has been reported that HFF-AL mice have altered diurnal patterns of behavior and food intake, suggesting that diet fat content can influence SCN activity (9, 56). To examine how the SCN was influenced by feeding regimens, we assessed immunostaining for c-Fos, a marker of neuronal activation. Initial statistical analysis by two-way ANOVA, revealed a significant main effect of feeding group [*F* (3, 21) = 20.84, *P* < 0.0001], time point [*F* (1, 21) = 31.26, *P* < 0.0001], as well as feeding group × time point interaction [*F* (3, 21) = 10.46, *P* < 0.0002]. Overt differences were seen in c-Fos expression in the SCN of rats fed a high-fat diet compared with those fed a standard diet at ZT3 (Tukey, *P* < 0.0001). In contrast, no overall difference was detected between HFF-AL and HFF-RF or between C-AL and C-RF SCN c-Fos expression (Fig. 2). As expected, C-AL and C-RF rats showed high SCN c-Fos levels at ZT3, which declined at ZT7 (Tukey, *P* < 0.002). In contrast, HFF-RF and HFF-AL animals displayed minimal levels of c-Fos expression in the SCN at both ZT3 and ZT7. Thus, RF does not appear to impinge on c-Fos expression in the SCN, whereas consumption of a diet dense in saturated fat leads to a significant dampening of SCN c-Fos expression.

The PVH is a key central component of the HPA axis, which may be implicated in FAA (13, 19, 32, 33). We next determined how c-Fos expression in the PVH varied according to the feeding regimen and time of day. Initial statistical analysis by two-way ANOVA, revealed a significant main effect of feeding group [*F* (3, 23) = 21.20, *P* < 0.0001], time point [*F* (1, 23) = 13.16, *P* < 0.001], as well as feeding group × time point interaction [*F* (3, 23) = 23.49, *P* < 0.0001]. Compared with the PVH of C-AL control rats, the PVH of C-RF rats displayed an increase in c-Fos in the premeal anticipatory phase at ZT3 (Tukey, *P* < 0.001). Furthermore, PVH c-Fos expression in C-RF rats declined in the postanticipation phase relative to controls (Tukey, *P* < 0.0001). By contrast, the PVH of C-AL rats increased c-Fos expression from ZT3 to ZT7 (Tukey, *P* < 0.02). Similar to the C-RF animals at ZT3, the PVH of HFF-RF rats showed higher c-Fos expression at ZT3 that was significantly greater than their HFF-AL counterparts, as well as that of C-RF and C-AL rats (Tukey, *P* < 0.0001). In HFF-RF rats, c-Fos expression in the PVH declined from ZT3 to ZT7 during postanticipatory phase, but remained significantly higher than that seen in the PVH of C-RF rats (Tukey, *P* < 0.0001). C-Fos expression within the PVH of HFF-AL rats was not significantly different than C-AL rats at the two time points inspected (Fig. 2).

The DMH is a central regulator of homeostasis, with studies suggesting that the pars compacta of this structure (DMHc) plays a key role in the expression of some forms of FAA. Initial statistical analysis by two-way ANOVA revealed a significant interaction of feeding group × time point on DMHc c-Fos levels [*F* (3, 21) = 9.49, *P* < 0.0004]. The DMHc of C-RF rats displayed a significant increase in c-Fos expression compared with C-AL controls at the ZT3 meal anticipatory time point (Tukey, *P* < 0.0001). Four hours later, postanticipation at ZT7, the DMHc of C-RF animals exhibited a decline in c-Fos expression (Fig. 2) compared with the ZT3 anticipatory time point. In contrast, the DMHc of C-AL animals had an increase in c-Fos expression at ZT7 relative to ZT3. HFF-RF and HFF-AL animals displayed lower c-Fos than C-RF rats at the ZT3 time point (Tukey, *P* < 0.01). Further, at this time point, c-Fos expression in the DMHc of HFF-RF rats did not differ from that of HFF-AL controls (Fig. 2). Four hours later at postanticipation phase, c-Fos levels in the DMHc of HFF-RF rats were comparable to those seen at ZT3. This observation suggests that unlike C-RF animals, c-Fos expression within DMHc of HFF-RF rats fails to entrain to the time of meal availability.

#### Augmentation and altered entrainment of plasma corticosterone in rats subject to daily restricted access to a high-fat diet.

**Fig. 3.**
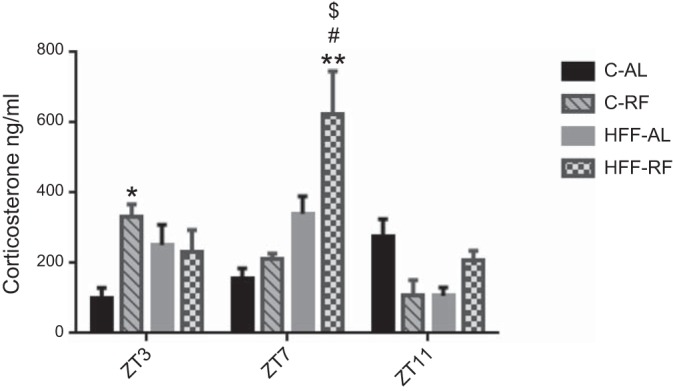
Effects of diet on the day-time profile of plasma corticosterone. With standard chow diet, restricted feeding leads to a prominent anticipatory rise in corticosterone compared with C-AL animals (ZT3). Postanticipation, corticosterone declines in the C-RF animals, whereas there is a prominent elevation of corticosterone in HFF-RF rats relative to other groups (ZT7). This declines at ZT11, whereas the C-AL rats show the typical prenocturnal rise in corticosterone. *P < 0.01, **P < 0.001 significant within diet comparison. $Significant compared with C-RF group. #Significant difference compared with C-AL group. *n* = 8 per group.

A premeal rise in corticosterone may be necessary for the occurrence of robust FAA. Since high-fat feeding alters corticosterone rhythms, we sought to compare the effect of C-RF and HFF-RF on the entrainment of plasma corticosterone. Initial statistical analysis revealed a significant main effect of feeding group [*F* (3, 84) = 6.148, *P* < 0.0008], time point [*F* (2, 84) = 9.217, *P* < 0.0002], as well as interaction of these two factors [*F* (6, 84) = 7.539, *P* < 0.0001]. C-AL rats had relatively low levels of plasma corticosterone, which gradually increased from ZT3 (100 ng/ml) to ZT7 (154 ng/ml) rising further by ZT11 (>270 ng/ml). In contrast corticosterone levels in HFF-AL rats rose from 251 ng/ml at ZT3 to 340 ng/ml at ZT7 and then reduced to 106 ng/ml at ZT11. Plasma corticosterone of C-RF rats was threefold higher at the anticipatory time point (Fig. 3) compared with C-AL rats (Tukey, *P* < 0.01). Postanticipation, C-RF plasma corticosterone dropped, and was comparable to levels seen in C-AL animals at ZT7. At the time of expected meal presentation, corticosterone levels in HFF-RF rats were of similar concentration to that of their HFF-AL counterparts. This suggests that unlike C-RF rats, their HPA axis had not reorganized in response to periodic availability of food. Surprisingly, HFF-RF corticosterone levels increased substantially in the postanticipatory phase, to reach 623 ng/ml at ZT7 (Fig. 3), which was significantly greater than levels observed in both HFF-AL rats (Tukey, *P* < 0.001), as well as C-AL and C-RF rats at this time point (Tukey, *P* < 0.0001). By ZT11, HFF-RF corticosterone fell to similar levels (∼206 ng/ml) as those observed in HFF-AL rats.

#### Differential adaptation of metabolism and behavior in rats given restricted access to standard and high-fat diet.

**Fig. 4.**
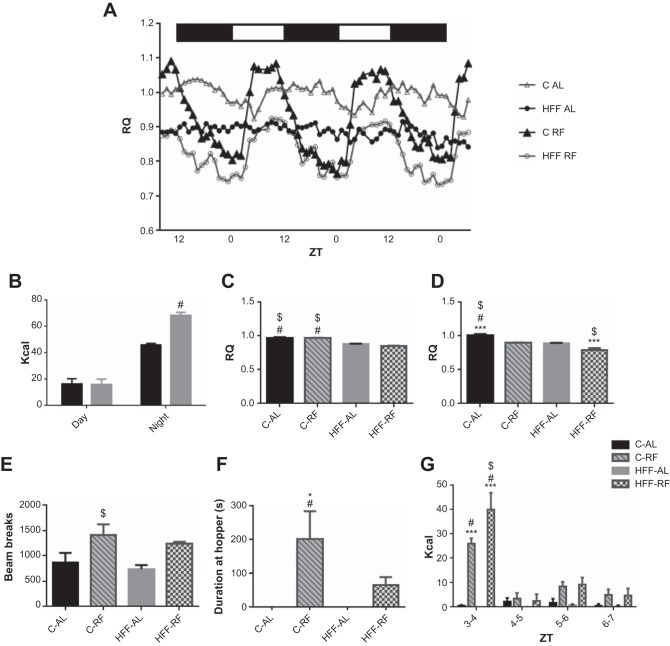
Effect of diet on the daily profile of respiratory quotient (RQ). *A*: smoothed averaged RQ traces from rats of the different feeding groups. Compared with the daily rhythm seen in C-AL rats, the HFF-AL rats exhibit a very blunted rhythm. Under restricted feeding, C-RF animals have a high-amplitude rhythm, whereas HFF-RF rats display a dampened rhythm. (△: C-AL; ●: HFF-AL; ▲: C-RF; and ○: HFF-RF). *B*: day-night variation in food intake was comparable between C-AL and HFF-AL rats. *C*: peak in daytime RQ was significantly reduced in HFF-AL and HFF-RF animals. *D*: compared with C-AL animals, night-time RQ was significantly reduced in HFF-RF rats. *E*: locomotor activity as recorded by total beam breaks over ZT1–ZT3 tended to be elevated in C-RF and HFF-RF animals. *F*: total time spent oriented to the food hopper over ZT1–ZT3 was significantly elevated in the C-RF, but not HFF-RF animals relative to ad libitum fed controls. *G*: HFF-RF rats consume significantly more calories than C-RF rats and control groups within the first hour of meal availability (ZT3–ZT4). No differences in calorie consumption were observed between groups over ZT 4–7 of the RF meal time window. *P < 0.01; ***P < 0.0001. *n* = 4–6 per group. # and $*P* < 0.01 compared with rats fed the counter diet on an ad libitum or RF basis, respectively.

Using indirect calorimetry cages, we initially assessed the impact of ad libitum access to the two different diets on day-night variation of food intake. Initial statistical analysis revealed a significant main effect of diet [*F* (1, 16) = 8.21, *P* < 0.01], time point [*F* (1, 16) = 113.6, *P* < 0.0001], and interaction of these two factors [*F* (1, 16) = 8.609, *P* < 0.009]. Post hoc tests revealed that both C-AL and HFF-AL animals significantly vary their food intake, such that they consume more calories during the night (both Tukey, *P* < 0.0001). In addition, HFF-AL rats consumed significantly more calories at night than C-AL counterparts (Tukey, *P* < 0.0001). These observations suggest that in the experimental setup adopted here, ad libitum access to a high-fat diet does not lead to increased day-time feeding activity. We next set out to explore how rats fed these diets made metabolic adaptations in response to restricted feeding. Relative to C-AL rats, constant access to the 45% high-fat diet caused a noticeable dampening of the of the diurnal RQ profile (Fig. 4). This raises the possibility that these HFF-AL animals are more active during the day; however, assessment of ambulatory activity (beam breaks) and time spent at the food hopper by HFF-AL animals did not indicate increased active behaviors relative to C-AL rats (*P* > 0.05; data not shown). Average RQ values for the light and dark phases were calculated for each feeding group. Initial statistical analysis revealed a significant main effect of feeding group on day [*F* (3, 13) = 40.67, *P* < 0.0001] and night RQ [*F* (3, 13) = 79.62, *P* < 0.0001] RQ values. Previous metabolic studies performed in C-RF animals report substantial reorganization of substrate utilization with a tendency for animals to store fat upon satiation (30, 49). Post hoc tests revealed that C-RF day-time RQ values (which include postsatiation) were constantly higher than those seen in HFF groups (Tukey, *P* < 0.001). Visual inspection of the RQ profiles of RF animals showed that RQ values of both C-RF and HFF-RF rats overtly drop during fasting. Once satiated, RQ rises gradually in HFF-RF and increases sharply in C-RF rats (Fig. 4). C-RF RQ was significantly higher than HFF-RF rats during both the 12 h of light (Tukey, *P* < 0.001) and 12 h of dark (Tukey, *P* < 0.001), reflecting the lower fat content of the diet. The averaged RQ values of HFF-RF animals declined from morning to night and were significantly lower than those of all other groups at the latter time point (Tukey, *P* < 0.001).

The indirect calorimetry chambers were then used to provide an objective measure of behavior. C-RF and HFF-RF total beam breaks were tallied into ZT time bins and plotted to give an overall analysis of cage activity during FAA (Fig. 4). Initial statistical analysis revealed a significant main effect of feeding group on premeal ambulatory cage movements [*F* (3, 8) = 4.63, *P* < 0.03]. Consistent with the initial behavioral observations in the first experiment, C-RF and HFF-RF rats show more ambulatory activity than their ad libitum counterparts during anticipation (ZT1–ZT3), but this did not reach statistical significance (Tukey, *P* > 0.05). However, C-RF rats displayed significantly more ambulatory cage activity than HFF-AL rats (*P* < 0.05). This confirms that general cage movements are a poor measure of FAA (44, 61). We next assessed the total number of beam breaks occurring in the premeal time window as an objective measure of meal anticipation. Initial statistical analysis revealed a significant main effect of feeding group on meal anticipatory hopper visits [*F* (3, 12) = 4.97, *P* < 0.01]. In agreement with the first experiment, C-RF rats spent significantly more time at the feeding hopper than C-AL and HFF-AL controls (Tukey, *P* < 0.01). In contrast, no significant difference was detected for the duration of time spent at the food hopper between ZT1 and ZT3 among HFF-AL and HFF-RF rats (Tukey, *P* > 0.05), indicating lower FAA in rats fed a daily meal of a high-fat diet.

We subsequently determined whether the pattern of food consumption differed between C-RF and HFF-RF rats. The total number of calories consumed by each feeding group between ZT3 and ZT7 was tallied into hourly time bins. Initial statistical analysis revealed a significant main effect of feeding group [*F* (3, 80) = 21.03, *P* < 0.0001], time point [*F* (3, 80) = 20.39, *P* < 0.0001], and interaction of these two factors [*F* (9, 80) = 8.41, *P* < 0.0001] on the pattern of food intake. Post hoc tests revealed that within the first hour (ZT3–ZT4) of meal availability, both RF groups significantly increased food consumption relative to ad libitum control groups (Tukey, *P* < 0.0001). In addition, HFF-RF rats consumed significantly more calories than C-RF counterparts within the first hour of meal presentation (Tukey, *P* < 0.001). No significant between-group differences in food consumption were detected at other hourly points in the RF time window.

#### Treatment with RU486 attenuates C-RF but enhances HFF-RF meal anticipation.

We generated a pharmacokinetic profile for RU486 a compound with potent antiglucocorticoid action (6, 65, 76). On the basis of the results obtained from the corticosterone profile, as well as evidence from the published literature (19, 33, 38, 40, 42, 79), it was hypothesized that blocking the action of corticosterone in HFF-RF rats would rescue FAA. A pharmacokinetic study was first performed to determine the clearance rates of orally and intravenously administered RU486 in male HW rats. We set out to gain a suitable dose of RU486, which when given to HFF-RF rats at ZT7, would antagonize the action of high-corticosterone levels for around 18 h, before permitting corticosterone action in the time just before the daily meal. Oral dosing with 10 mg/kg RU486 led to rapid clearance of the compound with the maximum concentration of 0.01 μg/ml observed 1 h after dose. Orally dosed RU486 is reported to achieve near-complete GR occupancy 2 h postadministration (65). Since the concentration of RU486 observed 2 h after oral dosing (0.01 μg/ml) could still be observed in the intravenously dosed rats 12 h postadministration, we postulated that GR occupancy is achieved for 12–18 h after intravenous dose (data not shown).

**Fig. 5.**
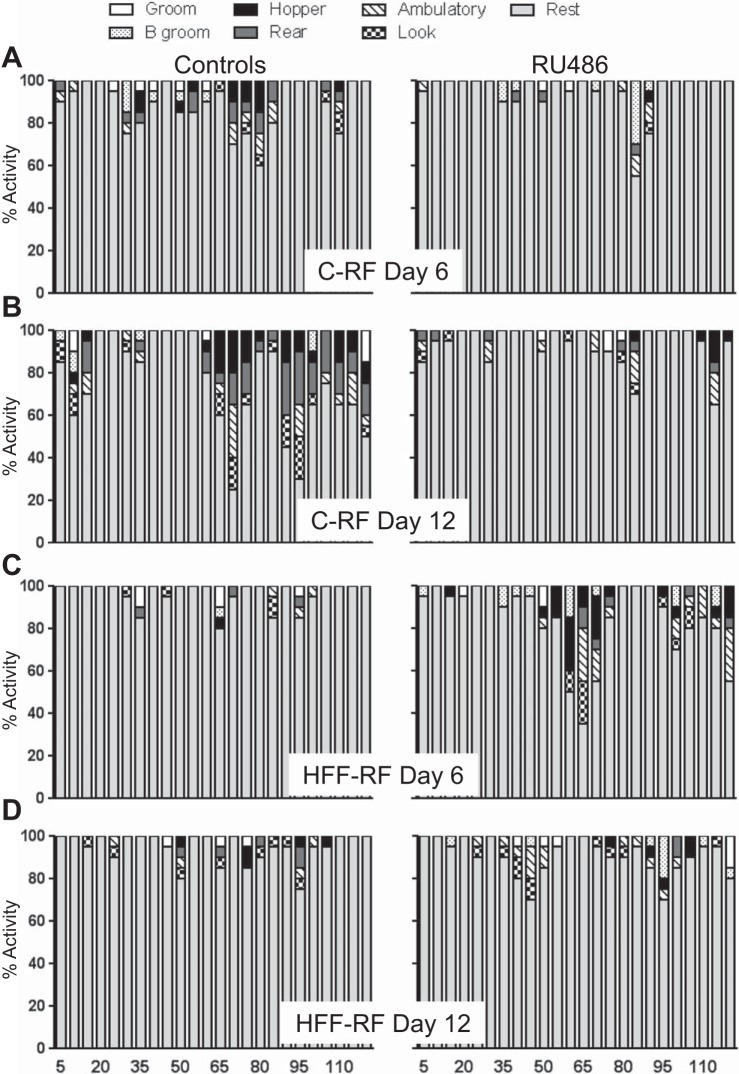
Corticosterone modulates meal-anticipatory behavior in restricted-fed rats. *A* and *B*: C-RF animals receiving vehicle showed the typical increase in meal anticipatory behavior (ZT1-3) over the 12 days of study. A daily injection of RU486, which antagonizes the action of corticosterone dramatically attenuated displays of this behavior in the premeal window. *C* and *D*: HFF-RF rats receiving vehicle showed infrequent expression of meal anticipatory behavior in the time preceding meal presentation. A daily injection with RU486 initially caused an apparent increase in meal anticipation in HFF-RF rats (*day 6*), which declined by *day 12*. C-RF rats receiving RU486 showed less meal anticipatory hopper visits and active behaviors than vehicle-injected controls (see Fig. 6). *n* = 4 per group.

It was also hypothesized that in C-RF rats, the rise in corticosterone in the anticipatory phase facilitates FAA, whereas the high corticosterone levels observed in HFF-RF rats prevents the occurrence of robust FAA. The final aim then was to determine whether FAA could be rescued in HFF-RF rats by timed antagonism of corticosterone action. To this end, C-RF and HFF-RF rats received a daily dose of vehicle (V) or RU486 (RU) for the duration of 12 days of restricted feeding. Initial observations revealed noticeable alterations in the expression of active behaviors in response to treatments that were different between rats receiving a low- and high-fat diet (Fig. 5). Given that treatment with the antiglucocorticoid RU486 would likely impinge upon energy homeostasis, we also assessed the effect of drug treatment on body weight. The effect of repeated daily dosing with 5 mg/kg RU486 on body weight was assessed by repeated-measures ANOVA, which revealed a significant main effect of study day [*F* (5, 60) = 5.78, *P* < 0.0002]. Tukey post hoc tests showed that this was due to the body weight of C-RF V rats being consistently lower than HFF-RF V rats from *day 4* until the end of the study (*P* < 0.01).

**Fig. 6.**
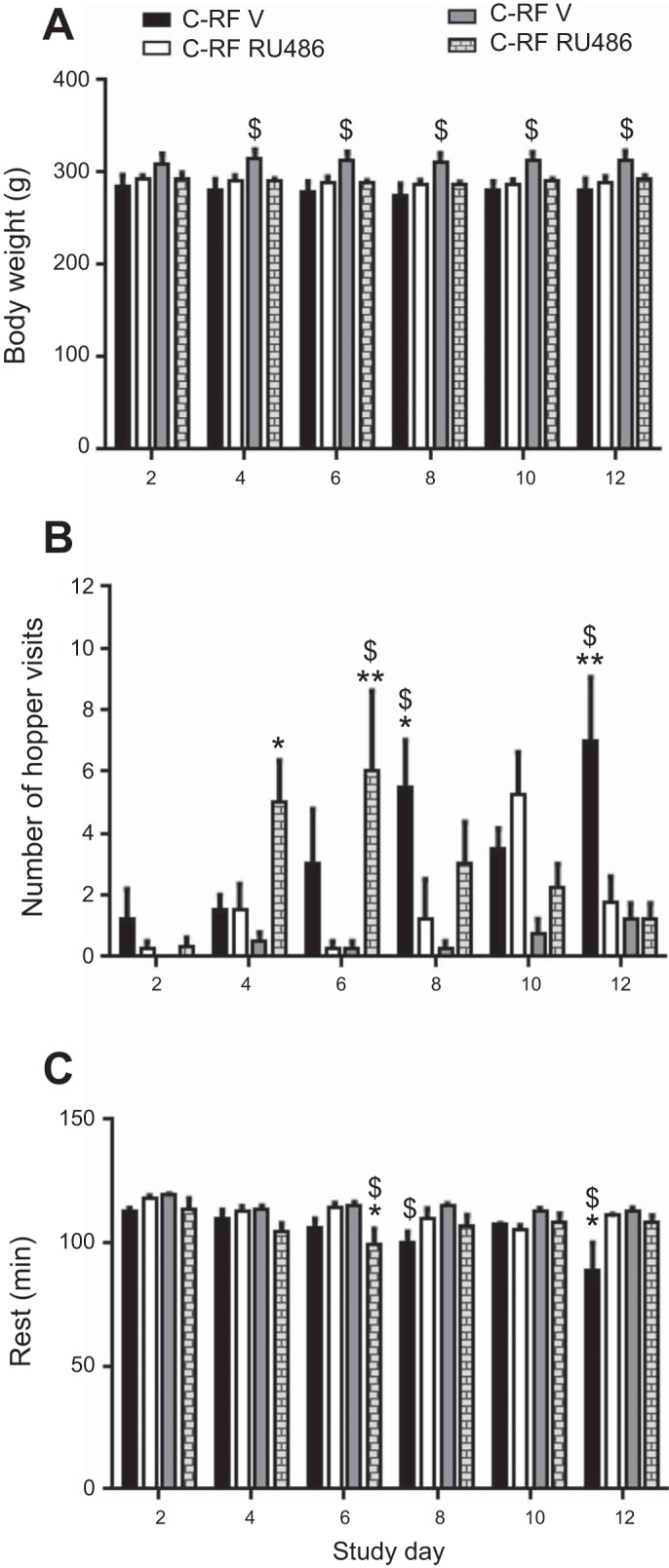
Treatment with RU486 has differential effects on meal anticipation in rats receiving low- and high-fat diet. *A*: compared with appropriate vehicle-treated controls, repeated daily dosing with RU486 did not significantly affect body weight. Instead, HFF-RF V rats were consistently heavier than C-RF V rats. *B*: from *day 8* onward, C-RF RU rats significantly reduced meal anticipation relative to C-RF V controls. Conversely, at *day 6*, HFF-RF RU-treated rats showed an increase in anticipation relative to HFF-RF V rats, but this was lost by *day 8. C*: C-RF V rats displayed a progressive decrease in resting behavior, which became significant compared with HFF-RF V rats by *day 8* and relative to all other groups by *day 12*. HFF-RF RU rats showed less resting behavior than C-RF RU and HFF-RF V rats on *day 6*, but were comparable to C-RF V rats on *day 6*. *P < 0.01, **P < 0.001, significant difference within diet comparisons. $Significant difference compared with RF group on counter diet. *n* = 4 per group.

Treatment with RU486 noticeably inhibited active behaviors in C-RF rats, but induced activity in HFF-RF rats. Initial statistical analysis revealed a significant main effect of feeding group [*F* (3, 12) = 6.451, *P* < 0.007], study day [*F* (5, 60) = 3.146, *P* < 0.01], and an interaction of study day × feeding group [*F* (15, 60) = 3.22, *P* < 0.0006] on premeal hopper approaches. As early as 4 days into restricted feeding, HFF-RF V rats showed significantly less hopper approaches than their HFF-RF RU486 counterparts (Tukey, *P* < 0.01). This trend continued until *day 6* with HFF-RF RU486 rats showing enhanced activity, which was comparable to C-RF V rats (Figs. 5 and 6) with statistically greater hopper approaches than HFF-RF V (Tukey, *P* < 0.001) and C-RF RU486 rats (Tukey, *P* < 0.001). This suggested that treatment with RU486 induced meal anticipation in HFF-RF rats but inhibited anticipation in C-RF rats up until this point in the study.

By *day 8*, the enhanced meal anticipation in HFF-RF RU486 rats was still apparent, but statistical significance was lost (Fig. 6). From *day 8* onward, C-RF V rats showed the tendency to display more active behaviors than C-RF RU486 (Tukey, *P* < 0.01) and HFF-RF V (Tukey, *P* < 0.001) rats. However, C-RF V rats were not statistically different from HFF-RF RU486 rats, suggesting some residual efficacy of drug treatment in maintenance of meal anticipation in the latter mentioned group. By the final day of recording, HFF-RF RU486 rats showed comparable activity profiles to that seen in vehicle controls, indicating loss of efficacy in this group. Conversely, C-RF V rats showed significantly more meal anticipatory hopper visits than all other groups (Tukey, *P* < 0.001). Thus, in response to RU486, HFF-RF rats increase meal anticipation, albeit transiently, while C-RF rats show a progressive decline in meal anticipation.

## DISCUSSION

Taken together, the current study shows that attenuated FAA seen in rats fed a high-fat diet is associated with altered expression of c-Fos in the DMHc, PVH, and SCN. In addition, the daily profile of corticosterone was changed in HFF-RF rats, with a large rise occurring during the postanticipatory period. This is in contrast to the premeal food anticipatory rise in corticosterone observed in C-RF rats. Use of the indirect calorimetry chambers offered another measure of the attenuated FAA measured by hopper approaches in HFF-RF rats, as well as lower RQ values. Interestingly, we show for the first time that blockade of corticosterone action by treatment with 5 mg/kg iv RU 486 attenuates FAA in C-RF rats, while enhancing FAA in HFF-RF rats at least temporarily. These results indicate that an acute rise in corticosterone is necessary for the onset of FAA, but a chronic elevation of corticosterone suppresses FAA.

Daily restricted access to a single meal is associated with the development of FAA, which can be measured by means of premeal rising body temperature, total cage activity, exercise or food-bin approaches, with the intensity of the given activity being measured, indicating degree of entrainment (55, 61). In agreement with previous studies, HFF-RF rats displayed attenuated FAA compared with C-RF rats (22, 70). Given the robust recovery of total calorie consumption observed in HFF-RF animals compared with C-RF rats, it is possible that C-RF rats were more food-restricted and, consequently, manifested elevated meal anticipation. However, despite the relative calorie deficit observed in C-RF rats, body weight is closely regulated, being comparable to C-AL controls. Further, RQ measurements showed that C-RF rats have a steep rise in RQ postsatiation, which reflects deposition of fat, the likely mechanism by which body weight is maintained in this group. Therefore, C-RF rats consume fewer calories but defend body weight, as evidenced by the comparable energy efficiency between feeding groups.

Given the possible role of the DMHc in contributing to some forms of FAA and the reported rise of c-Fos expression in this structure in meal-anticipating rats, we measured the induction of c-Fos expression in the DMHc (3, 26, 45, 47). In agreement with this, C-RF rats in the current study displayed a significant increase in DMHc c-Fos expression relative to controls, while HFF-RF rats failed to show this anticipatory rise in DMHc c-Fos expression. This observation supports a role for activation of the DMH in the expression of FAA.

The PVH is the site of corticotrophin-releasing-factor production, a contributing factor to the HPA axis. Although some studies suggest that this structure is not necessary for FAA, it may be of importance to meal anticipation through entrainment of corticosterone rhythms (19, 46, 59). In the current study, an anticipatory rise in PVH c-Fos expression was observed for C-RF and to a greater extent HFF-RF rats, despite the lack of FAA in the latter group. This observation highlights the importance of lesion studies in the search for the FEO. At ZT3 we observed a particularly high level of c-Fos expression in the PVH of HFF-RF rats, which potentially reflects high corticotrophin-releasing factor production at this time point. This may then have contributed to the postanticipatory rise in corticosterone detected in HFF-RF rats at ZT7.

Restricting feeding time rather than overt restriction of calories does not appear to affect SCN function (11, 16). In agreement with this, the C-RF regimen had no noticeable effect on SCN c-Fos expression compared with C-AL rats. In contrast, high-fat feeding under ad libitum or restricted feeding conditions suppressed the expression of c-Fos in the SCN at the time points inspected. This observation supports those studies reporting altered SCN molecular synchronization to light following long-term high-fat feeding in mice (56). The indirect calorimetery study showed that HFF-AL rats have a similar diurnal pattern of behavior and feeding to that of C-AL counterparts. This is surprising when one considers the SCN c-Fos results, as well as previous reports of increased day time activity and feeding in mice fed a high-fat diet, but it may reflect the different experimental setup used here (40).

Consistent with previous reports (9, 79), HFF-AL rats had increased plasma corticosterone levels during the early light phase time points examined, but significantly lower corticosterone shortly before the dark phase. The SCN directly innervates the corticotrophin-releasing factor-producing neurons of the PVH (81) and may, therefore, exert circadian regulation over the HPA axis by inhibiting activity of the PVH neurons by its timed secretion of vasopressin into the PVH (1, 8, 36). The decreased SCN c-Fos expression in the early light phase of animals under HFF-AL/HFF-RF conditions could, thus, result in disinhibition of the PVH. This is a possible mechanism for the increased HPA axis and resulting circulating corticosterone levels observed in these animals. Further, within the raphe nuclei of the brain stem, the rhythmic expression of tryptophan hydroxylase 2, the rate-limiting enzyme for 5HT production, is dependent upon an intact corticosterone rhythm (51). The raphe nuclei send dense 5HT-containing projections to the SCN. In response to nonphotic, phase-shifting stimuli, the raphe releases 5HT into the SCN that can inhibit c-Fos expression (4, 24, 25). Thus, it is plausible that the increased corticosterone levels resulting from high-fat feeding have an impact on SCN function and output.

Some studies have shown that a premeal rise in plasma corticosterone recurs daily during prolonged starvation, supporting the contention that this hormone has an important role in FAA (32, 33), although recent reports indicate otherwise (69, 74, 78). Duclos et al. (19) demonstrated a loss of FAA in ADX rats, an effect reversed by daily premeal corticosterone injections, which corroborated earlier observations (15, 19, 32, 33). However, a more recent study showed that ADX-RF rats receiving an evening injection of corticosterone in anti-phase to the daily meal time, eventually develop FAA, suggesting that cues other than corticosterone can enable FAA (78). Further, clock gene protein rhythms in limbic brain structures and metabolic organs, such as the liver, were shown to entrain to feeding independent of corticosterone, suggesting that this hormone is not necessary for some aspects of meal anticipation (74, 78). In the current study, C-RF entrainment of the HPA axis was associated with a premeal rise in corticosterone at the expected meal time, with levels falling postanticipation in the absence of food intake. Conversely, HFF-RF rats failed to display an anticipatory rise in corticosterone above levels seen in HFF-AL rats, but instead showed a robust postanticipatory rise in corticosterone.

Considerable published evidence suggested to us that targeting corticosterone action within a specific time frame could rescue HFF-RF FAA. To begin, a number of animal models possessing elevated HPA axis activity, such as the diet-induced obese rat (79), F344 rat (18), and rats receiving high doses of corticosterone (19) all share a common trait of attenuated FAA. Thus, although intact adrenal glands and an anticipatory rise of corticosterone can contribute to robust FAA (19), corticosterone excess may inhibit FAA in HFF-RF rats (22). On the basis of the pharmacokinetic profile of intravenously administered RU486 (5 mg/kg), we extrapolated that corticosterone action would be broadly blocked for the 16 h following dosing. Further, the dose administered at ZT7 was postulated to leave a window of corticosterone action prior to presentation of the meal at ZT3, thereby permitting meal anticipatory active behaviors (65).

Within 6 days of repeated daily dosing of C-RF rats with RU486, there was a progressive attenuation of FAA, as exemplified by increased rest and decreased hopper, approaching of C-RF RU486 compared with vehicle-treated C-RF rats. Such findings support the notion that corticosterone action is required for normal FAA. In contrast, repeated daily dosing of RU486 initially increased FAA of HFF-RF rats to levels observed in vehicle-treated C-RF rats. However, this effect declined in the latter part of the experiment. This loss of action of RU486 may have been due to the buildup of this compound, thereby evoking long-term antagonism of GRs throughout the premeal time window. In addition, the HPA axis adaptation (desensitization and alterations in receptor and hormone expression), may contribute to this loss of efficacy in HFF-RF animals treated with RU486 (15, 23, 65). However, there are inconsistencies with this explanation since C-RF rats treated with RU486 exhibit reduced meal anticipation by *day 6* (relative to vehicle-treated C-RF controls), while HFF-RF animals treated with RU486 do not. This occurs prior to the loss of efficacy of RU486-treated HFF-RF rats, suggesting that accumulation of RU486 over time is unlikely to fully account for this loss of efficacy.

### Perspectives and Significance

In summary, attenuated FAA in HFF-RF rats is associated with decreased DMHc c-Fos expression, increased PVH c-Fos, and a failure to manifest an anticipatory rise in plasma corticosterone. Instead, HFF-RF rats present a marked postanticipatory rise in corticosterone, which when antagonized within a specific time frame rescues FAA. Our findings suggest that elevated basal corticosterone that can accompany obesity acts to suppress behavioral and physiological adaptation to restricted feeding regimens. Interestingly, human high waist-to-hip ratio forms of obesity are a high risk group for diabetes and are typified by a “cortisol response” to lunch-time meals (41, 73). It is interesting to speculate that this cortisol response is a human manifestation of the post-anticipatory corticosterone in HFF-RF rats observed herein; in such a case, anti-obesity drugs targeting the HPA axis may benefit from utilizing the HFF-RF animal model. Furthermore, our findings support the notion that a premeal rise in corticosterone promotes FAA in C-RF rats.

## GRANTS

This research was supported by project grant funding from the Biotechnology and Biological Sciences Research Council (BBSRC) to Hugh Piggins (grant no. BB/G004307), a BBSRC CASE studentship to Sara Namvar, and by operating funds from AstraZeneca.

## DISCLOSURES

No conflicts of interest, financial or otherwise, are declared by the authors.

## AUTHOR CONTRIBUTIONS

Author contributions: S.N., A.G., B.L., and H.D.P. conception and design of research; S.N., A.G., and M.D. performed experiments; S.N. and M.D. analyzed data; S.N., B.L., and H.D.P. interpreted results of experiments; S.N. and H.D.P. prepared figures; S.N. drafted manuscript; S.N., A.G., B.L., and H.D.P. edited and revised manuscript; S.N., A.G., M.D., B.L., and H.D.P. approved final version of manuscript.
